# Pharyngeal spreading of peri-implant infections under antiresorptive/antiangiogenic therapy

**DOI:** 10.1186/s40729-021-00332-z

**Published:** 2021-06-03

**Authors:** Karsten Kern, Fania Lukmann, Karina Obreja, Sara Al-Maawi, Bellinghausen Carla, Shahram Ghanaati, Gernot Rohde, Robert Sader, Frank Schwarz

**Affiliations:** 1 Department of Oral Surgery and Implantology, Goethe University, Carolinum, Frankfurt, Germany; 2 Department of Neurology, Knappschaftskrankenhaus, Sulzbach, Germany; 3 Department for Oral, Cranio-Maxillofacial and Facial Plastic Surgery, Medical Center of the Goethe University Frankfurt, Frankfurt, Germany; 4 Department of Respiratory Medicine and Allergology, University Hospital, Goethe University, Frankfurt, Germany

**Keywords:** Animal experiment, Peri-implant infections, Antiresorptive/antiangiogenic therapy, Immunohistochemistry, CD68 antigen

## Abstract

**Objectives:**

To assess the influence of antiresorptive/antiangiogenic therapy on the spreading of peri-implant infections in the pharyngeal region.

**Material and methods:**

This analysis was based on tissue biopsies obtained from a total of twenty-five albino rats having either received (1) amino-bisphosphonate (Zoledronate) (Zo) (*n*=4), (2) RANKL inhibitor (Denosumab) (De) (*n*=4), (3) antiangiogenic medication (Bevacizumab) (Be) (*n*=4), (4) Zo+Be (*n*=3), (5) De+Be (*n*=5), or (6) no medication (Co) (*n*=5). Drug administration was repeated at 12 weeks. Chronic-type peri-implant infections were induced at titanium implants located in the upper jaws. The surface area (%) of infiltrated connective tissue (ICT) and CD68-positive cells was assessed within the lateral pharyngeal/retropharyngeal connective tissue zone.

**Results:**

Mean (±SD) and median ICT% values and CD68 counts were markedly highest in the De+Be (11.10±6.04; 11.81; 95% CI − 3.89; 26.11) and De (5.70±5.06; 6.19; 95% CI − 2.34; 13.75) groups, reaching statistical significance for De CD68 counts over the Co (0.18±0.25; 0.18; 95% CI −2.14; 2.51) group. In both De+Be and De groups, the ICTs were occasionally associated with an ulceration of the epithelial compartment.

**Conclusions:**

Induced peri-implant infections were not associated with any inflammatory lesions in pharyngeal tissues. While these findings were similar under Zo and Be medication, De and De+Be had a marked effect on ICT and CD68 values. The clinical relevance of these adverse findings needs further investigation.

## Introduction

Peri-implantitis is defined as a plaque-biofilm-related pathological condition occurring in tissues around dental implants. It is characterized by inflammation in the peri-implant connective tissue and a progressive loss of supporting bone [1]. The associated lesions are dominated by plasma cells and lymphocytes, thus sharing many similarities with those noted at periodontitis sites. In contrast to the latter, however, the inflammatory lesions at peri-implantitis sites were reported to be lager in size and revealed larger proportions of polymorphonuclear leukocytes and macrophages [2, 3].

Previously, peri-implantitis has also been linked with the occurrence and subsequently the pathogenesis of a medication-related osteonecrosis of the jaw (MRONJ) [4]. A potent drug is the amino-bisphosphonate zoledronic-acid (Za), which is gaining importance in the therapy of osteoporosis due to its long-lasting effect on increases in bone mineral density after intraveneous application [5]. On the contrary, however, the pro-inflammatory effects of Za were linked with acute phase reactions or orbital inflammation [6–8].

An increasing number of MRONJ cases are not only related to bisphosphonates, but also other antiresorptive (i.e., inhibitors of Receptor Activator of NF-κB Ligand (RANKL)) and antiangiogenic therapies [9–12]. While the inhibition of RANKL has a desired effect on the osteoclastogenesis in patients affected by bone-related diseases, this drug has also a potential influence on the host inflammatory response, since it may affect immune cells such as T cells, B cells, or monocyte–macrophages [13, 14]. All these findings seem to indicate that antiresorptive therapy may have the potential to influence the spreading of chronic oral infections such as peri-implant diseases. A common pathway for the spreading of odontogenic infections originating from either the maxilla or the mandible is the pharyngeal space, thus rendering this anatomical region as highly relevant [15]. Therefore, the aim of the present experimental study was to assess the influence of various types and combinations of antiresorptive/antiangiogenic medications on the spreading of peri-implant infections into the pharyngeal region in a rat model.

## Material and methods

### Animals

Forty-eight albino rats of the Wistar strain (age 6 months, mean weight 476±0.5 kg) obtained from a certified breeder were used in the study. All animals were housed in appropriately dimensioned cages under standard conditions of temperature in a light-controlled environment and were provided water and special diet ad libitum. The study protocol was approved by the appropriate local authority (Regierungspräsidium Darmstadt, Germany).

The present reporting followed the ARRIVE Guidelines [16].

### Study design and surgical procedures

**Fig. 1:**
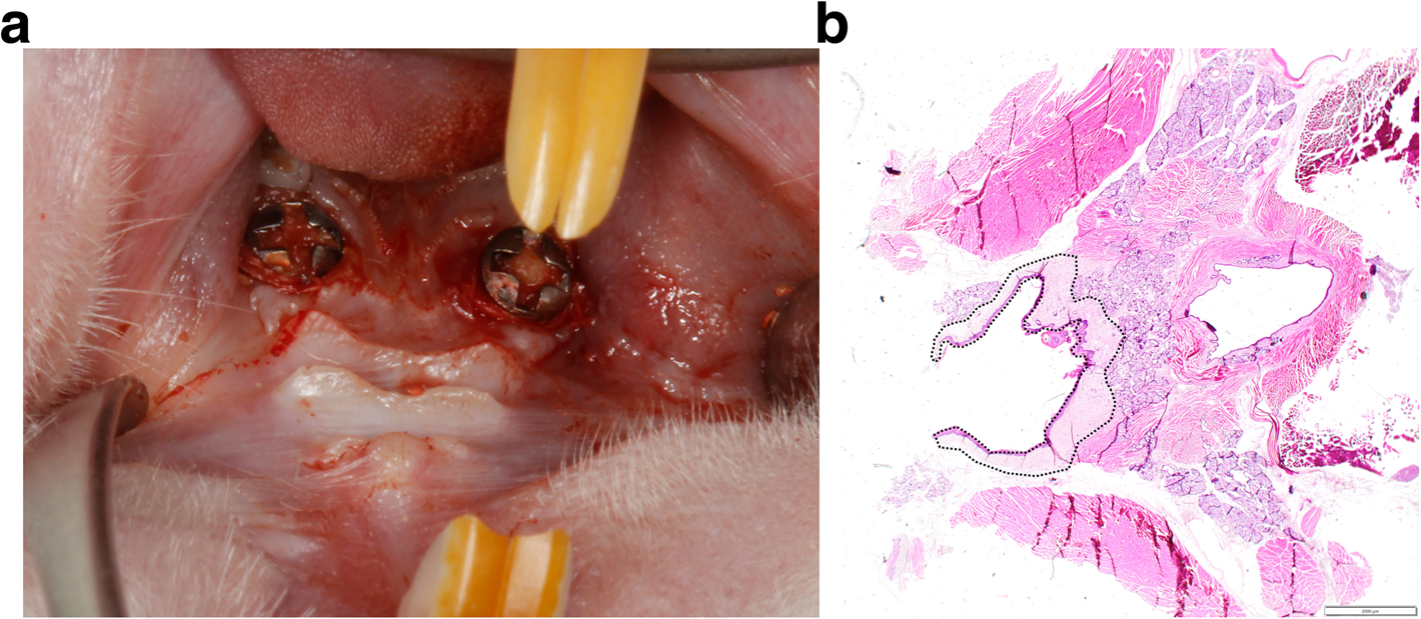
**a** Chronic-type peri-implant infections were induced at titanium implants placed in the upper jaw of rats. Bleeding on probing pointing to clinical signs of inflammation at ligature removal. **b** Depiction of the region of interest (i.e., the inner and outer confines of the lateral pharyngeal and retropharyngeal connective tissue zone) in the horizontal plane for the histological analysis

Following the extraction of both maxillary first molars, smooth-surfaced titanium mini-implants (Ustomed® Micro-Screws, Cross, ⌀ 1,2 mm, shortened to 2–3 mm) were inserted at respective sites and left to heal for 6 weeks. Subsequently, the animals had randomly received the following commonly applied antiresorptive/antiangiogenic medications, including *n*=8 animals each: (1) amino-bisphosphonate (Zoledronate 5mg/kg intravenous, Ribometa® 4mg/5ml, Hikma Pharma, Gräfelfing, Germany) (Zo), (2) RANKL inhibitor (Denosumab 60mg/kg subcutaneous, Prolia®, Amgen, Munich, Germany) (De), (3) antiangiogenic medication (Bevacizumab 5mg/kg intravenous, Avastin® 400 mg/16 ml, Roche Pharma, Grenzach-Wyhlen, Germany) (Be), (4) Zo+Be, (5) De+Be, or (6) no medication serving as control group (Co). Drug administration was repeated at 12 weeks. Subsequently, peri-implantitis lesions were induced by an established and validated procedure [17]. This included an intraperitoneal booster lipopolysaccharide (Lipopolysaccharide *Escherichia coli* O111:B4, EMD Millipore, Merck, Darmstadt, Germany) injection along with daily topical injections in the peri-implant sulcus at each implant site for 3 days. Subsequently, miniature polyester ligatures (6-0) were placed in a submarginal position around both implants in each animal for 4 weeks [18]. In brief, ligatures were forced into a position directly apical of the mucosal margin. Subsequently, a “pocket” was created to facilitate the establishment of a submucosal microflora. This was followed by a progression period of 12 weeks (Fig. 1a).

### Anesthesia protocol

For each surgical intervention, the animals were anesthetized by intraperiotoneal injection of 7.5 mg/kg ketamine (Ketanest®, Pfizer Pharma GmbH, Karlsruhe, Germany) and 5 mg/kg xylazine (Rompun®, Bayer HealthCare, Leverkusen, Germany). For postoperative analgesia, 4.5 mg/kg carprofene was administered subcutaneously immediately after surgery, as well as 1, 2, and 3 days postoperatively.

### Histological and immunohistochemical processing

The animals were euthanized with an overdose of pentobarbitone at 100 mg/kg. The pharyngeal space was dissected from intraorally and included the mucosal and submucosal tissue compartments (Fig. 1b).

The samples were decalcified using ultrasound supported water bath and Ethylenediaminetetraacetic (EDTA) for 1 week prior to processing and embedding in paraffin. Two sections of each block were cut in the horizontal plane with the micrometer set at 3–4 μm. One section was stained with hematoxylin and eosin (HE). The second section was used for immunohistochemical staining for cluster of differentiation 68 (CD68). After dewaxing in xylene, rehydration by a graded alcohol series, the samples were incubated in antigen retrieval solution (citrate buffer, pH=6) for 20 min in 96°C. Next, the sections were stained using a Lab VisionTM Autostainer 360-2D instrument (ThermoFisher Scientific, Germany) as described previously [19]. A primary mouse anti-rat monoclonal antibody was used to stain CD68 (dilution 1:1000, Bio-rad, MCA341R USA) for 30 min. Sections were washed with TBS buffer and positively stained cells were visualized using UltraVision™ Quanto Detection system HRP DAB (ThermoFisher Scientific, Germany). For negative controls, the primary antibody was replaced with non-immune serum.

### Histological and histomorphometrical analysis

Digital images (original magnification x 200; BX53, Olympus, Hamburg, Germany) were obtained from each specimen and evaluated using a software program (cellSens, Olympus).

In all sections, the inner and outer confines of the lateral pharyngeal and retropharyngeal connective tissue was delineated and defined as total surface area (TA). Within TA, the cross-sectional surface area and respective percentage of infiltrated connective tissue (ICT), as evidenced by the presence of CD68-positive cells, were assessed. Moreover, at 5 randomly selected regions of interest within TA but outside the demarcated ICTs, the total number of CD68-positive cells was counted (Fig. 1b).

All measurements were performed by one previously calibrated examiner. Calibration was accepted when repeated measurements of *n*=5 different sections were similar at >95% level.

Adequately preserved, intact pharyngeal tissue sections allowing a reproducible depiction of TA could be obtained from a total of twenty-five animals (Zo: *n*=4; De: *n*=4; Be: *n*=4; Zo+Be: *n*=3; De+Be: *n*=5; Co: *n*=5). The sections prepared from the remaining animals revealed an incomplete TA and were therefore not considered for the present analysis.

### Statistical analysis

The statistical analysis of the data sets was performed using a commercially available software program (IBM SPSS Statistics 27.0, IBM Corp., Armonk, NY, USA).

## Results

### Histomorphometrical analysis

**Fig. 2:**
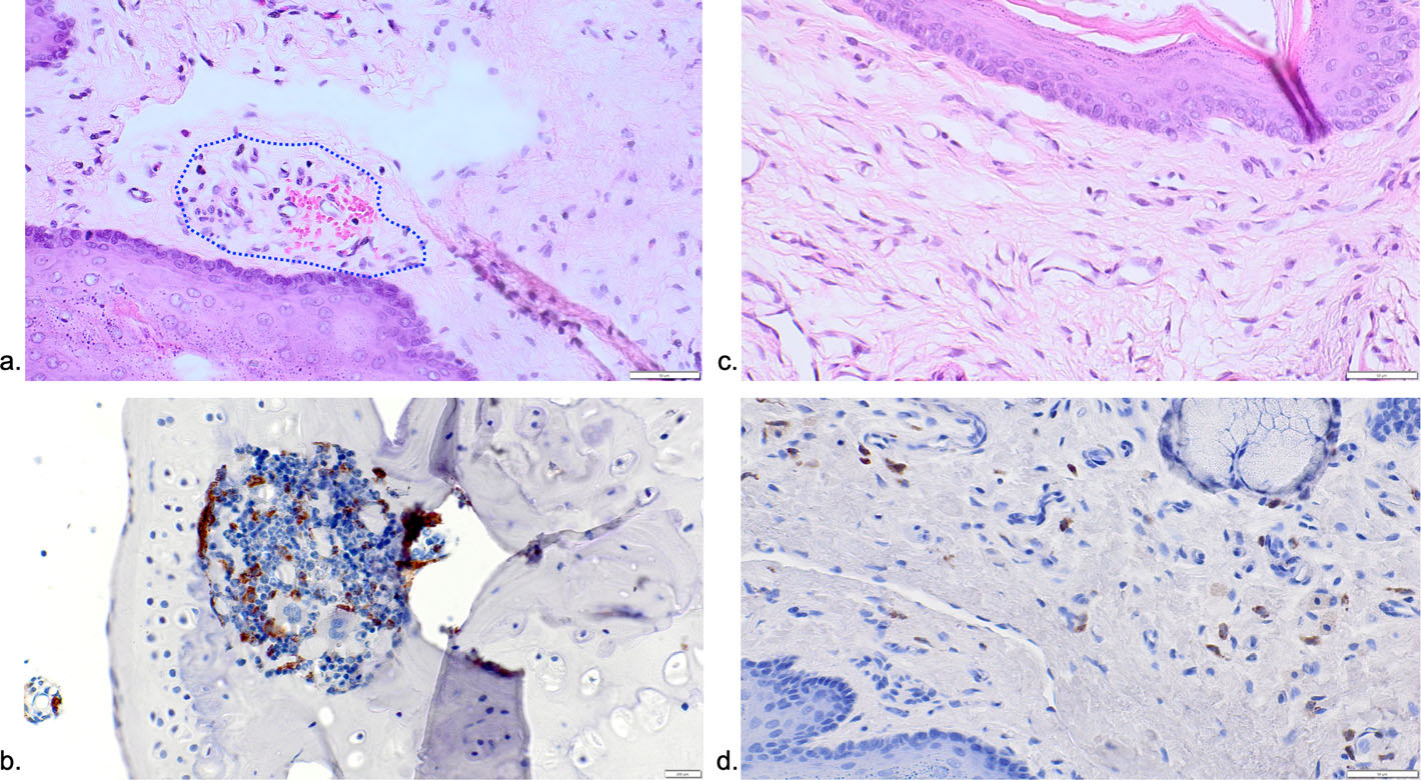
Representative histological and immunohistochemical views of tissue reactions in the Zo and Zo+Be groups. **a** Dotted area depicting the only specimen of the Zo group showing a mixed chronic inflammatory cell infiltrate in the subepithelial connective tissue compartment (Zo group, HE stain). **b** The ICT was commonly composed of CD68-positive cells, lymphocytes, polymorphonuclear leukocytes, and plasma cells (Zo group). **c** None of the biopsies from the Zo+Be group revealed any detectable ICT areas (HE stain). **d** Non-infiltrated connective tissue zone with single CD68 counts (Zo+Be group)

**Fig. 3:**
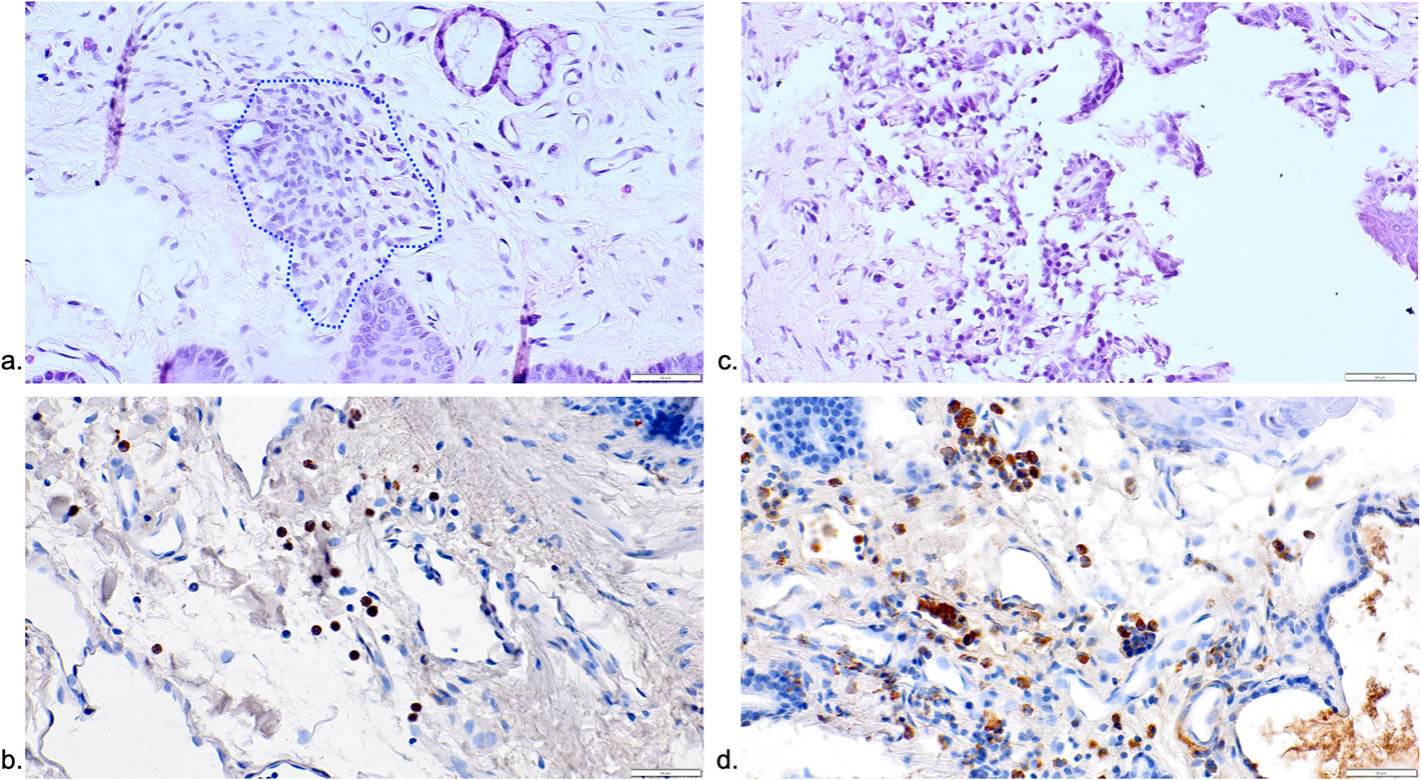
Representative histological and immunohistochemical views of tissue reactions in the De and De+Be groups. **a** De specimens commonly presented with well discernible ICT areas that were larger in size (HE stain). **b** A higher frequency of CD68-positive cells was also commonly noted outside the ICT areas (De group). **c** An infiltration of the epithelial compartment associated with a surface ulceration was occasionally noted in both De groups (De+Be group). **d** Specimen showing a massive infiltration of the connective tissue compartment with CD68-positive cells (De+Be group)

**Fig. 4:**
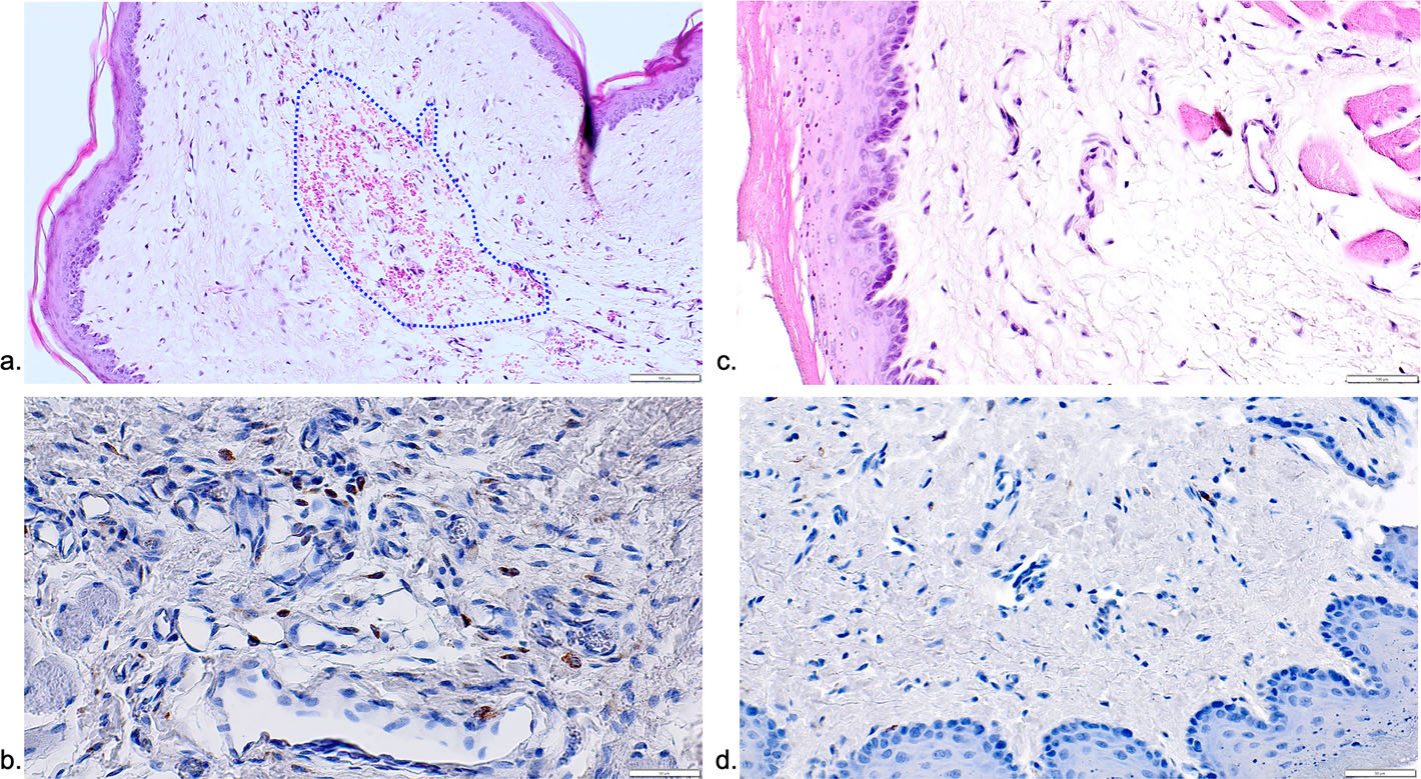
Representative histological and immunohistochemical views of tissue reactions in the Be and Co groups. **a** The frequency and extend of ICTs in the Be group was commonly very low (HE stain). **b** Only a few connective tissue areas presented with CD68-positive cells (Be group). **c** Specimens of the Co group mainly featured a non-infiltrated connective tissue zone. **d** The frequency of CD68-positive cells was commonly low (Co group)

Representative histological views of the lateral pharyngeal and retropharyngeal space in different groups are presented in Figs. 2, 3, and 4.

**Fig. 5:**
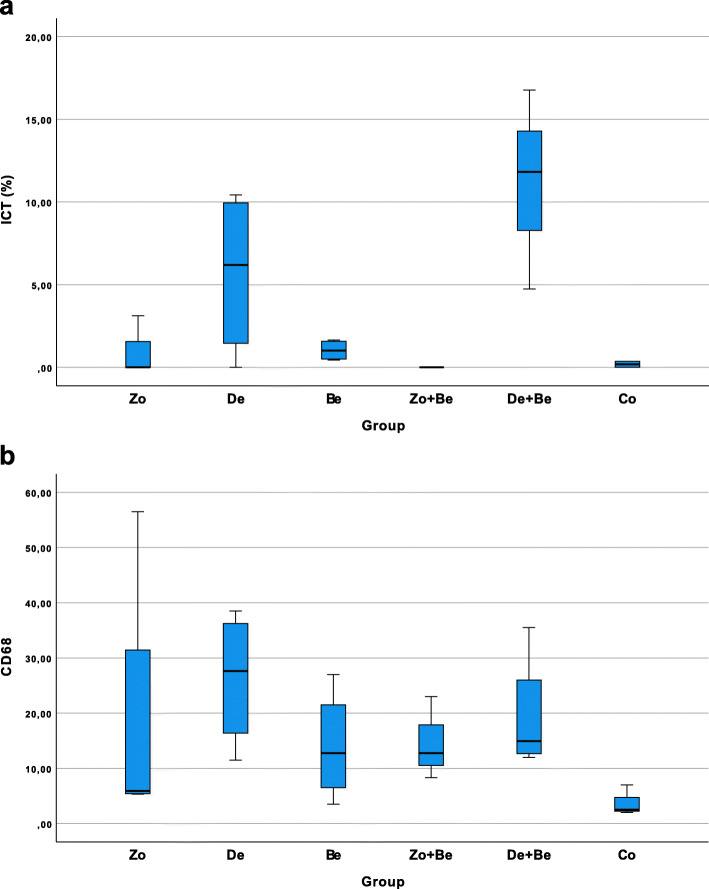
Boxplots to depict the histomorphometrical analysis in different groups (*n*=26 animals). **a** ICT% values. **b** CD68 counts. A significant difference was noted between De and Co groups (*P*=0.029; unpaired *t*-test)

Boxplots depicting the histomorphometrical analysis of ICT% and CD68 values in different groups are presented in Fig. 5a and b.

Mean (±SD) and median ICT% values were highest in the De+Be (11.10±6.04; 11.81; 95% CI −3.89; 26.11) and De (5.70±5.06; 6.19; 95% CI −2.34; 13.75) groups. This was followed by the Be (1.03±0.62; 1.02; 95% CI 0.03; 2.03) and Zo (1.04±1.80; 0.00; 95% CI −3.43; 5.51) groups. The lowest ICT% values were noted in the Co (0.18±0.25; 0.18; 95% CI −2.14; 2.51) group, whereas the Zo+Be group was associated with an absence of any noticeable ICT areas (Fig. 5a). Similarly to the latter, ICT areas in the Zo group were just noted in one of the specimens investigated (Fig. 2a–d).

The differences between the Zo (*P*=1.000; Mann–Whitney *U* test), De (*P*=0.220; unpaired *t*-test), Be (*P*=0.152; unpaired *t*-test), Zo+Be (*P*=0.423; unpaired *t*-test), De+Be (*P*=0.094; unpaired *t*-test) and Co groups, however, failed to reach statistical significance, respectively.

The respective ICT areas revealed a comparable composition of CD68-positive cells, but also lymphocytes, polymorphonuclear leukocytes, and plasma cells.

In all groups investigated, CD68-positive cells were markedly less frequent outside the ICT areas. Nevertheless, mean (±SD) and median CD68 values were also highest in the De (26.31±12.28; 27.62; 95% CI 6.76; 45.85) and De+Be (19.33±10.94; 14.91; 95% CI 1.92; 36.74) groups. This was followed by the Zo+Be (14.69±7.52; 12.75; 95% CI −3.99; 33.38), Be (14.00±10.05; 12.75; 95% CI −2.00; 30.00), and Zo (18.41±25.39; 5.91; 95% CI −21.98; 58.82) groups (Fig. 5b). The lowest CD68 values were noted in the Co (3.83±2.75; 2.50; 95% CI −3.00; 10.67) group, reaching statistical significance over the De (*P*=0.029; unpaired *t*-test) group. In both De and De+Be groups, ICTs including CD68-positive cells also occasionally invaded the epithelial compartment, thus leading to histological signs of surface ulcerations (Fig. 3a–d). In contrast, specimens of the Be and Co groups commonly revealed a well discernible and intact squamous epithelium and a loosely structured connective tissue compartment (Fig. 4a–d).

The regression analysis pointed to a positive correlation between CD68 and ICT% values, which, however, failed to reach statistical significance (*R*^2^ = 0.091; *B* = 0.302; *p* = 0.224).

## Discussion

The present study aimed at assessing the influence of various types and combinations of antiresorptive/antiangiogenic medications on the spreading of peri-implant infections into the pharyngeal region. The induction of chronic-type peri-implant infections followed an established and validated procedure and the resulting inflammatory lesions were shown to be associated with clinical signs of inflammation and a progressive loss of the implant supporting bone [17, 18].

The present results have indicated that mean and median ICT% values and CD68 counts were markedly highest in the De+Be and De groups, reaching statistical significance for De CD68 counts over the Co group. In contrast, Zo, Zo+Be, and Be groups were not commonly associated with any discernible ICT areas in the subepithelial connective tissue zone. Similar results were also noted for CD68 counts outside the ICT area, which were basically within the range of those noted in the Co group.

When further analyzing the characteristics of the ICTs, it was observed that they were dominated by CD68-positive cells, lymphocytes, polymorphonuclear leukocytes, and plasma cells. Basically, these characteristics closely resemble those commonly noted for mixed inflammatory cell infiltrates in peri-implantitis lesions [2, 3, 20–23]. As opposed to periodontitis lesions, the inflammatory lesions at implant sites were reported to be larger in size and associated with higher proportions of macrophages [2, 24]. Due to their potential relevance in the pathogenesis of peri-implant diseases [20], the present analysis had a major focus on the assessment of CD68-positive cells. In this context, it must also be emphasized that peri-implantitis lesions were characterized by an increase in the density of vascular structures [2], and that a marked portion of associated inflammatory cells were also present in peri-vascular compartments [3]. These circumstances may contribute to a potential spread of infections caused by a translocation of associated pathogens or endotoxins [25]. The latter mechanism has been described for periodontopathogenic bacteria having the potential to cause disseminating, non-oral infections [26]. Due to the close proximity to the oral cavity, the present analysis had a primary focus on the pharyngeal region.

A potential methodological drawback was the lack of microbiological analyses to identify associated bacteria in the evaluated tissue compartments. However, since the microbial picture associated with peri-implant diseases is currently regarded as incomplete and opportunistic pathogens, fungal organisms and viruses may also contribute to these rather complex and heterogeneous infections [1]; it may be challenging to specify a direct translocation of associated pathogens in future studies.

Apart from a direct transmission of pathogens, the presence of ICTs might also be linked with a systemic infection induced by the local inflammatory infiltrate [25]. In fact, disseminated pathogens can induce the secretion of pro-inflammatory cytokines by, e.g., leucocytes or endothelial cells but also a direct formation of immune complexes [27, 28]. These findings could not be confirmed when evaluating the present analysis of the Co group. Despite the lack of an additional control group (i.e., healthy peri-implant tissues, no drug administration), the absence of any noticeable ICT areas and low CD68 counts in the evaluated tissue biopsies of the Co group may point to a very limited potential of induced peri-implantitis lesions to spread in the pharyngeal region. This potential, however, appeared to be increased under De and De+Be medication. The noted marked increases in ICT values and CD68 counts as well as the noted epithelial ulcerations may be attributed to De and its potential to affect immune cells such as T cells, B cells, or monocyte–macrophages [13, 14]. This is supported by the findings of systematic reviews and meta-analyses also pointing to the potential of De to increase the risk of infections, mainly affecting ear, nose, and throat, but also the urinary tract and skin [29, 30]. Due to the lack of a corresponding negative control (i.e., healthy peri-implant tissues, De/De-Be administration), it is however difficult to estimate whether the present findings in both De groups have a causal association with the induction of peri-implant infections, or are in coincidence with drug administration. In this context, it must also be kept in mind that the number of tissue biopsies was particularly limited in the De+Be group, thus potentially affecting the statistical power of the analysis. While the clinical relevance of the present findings is unclear, future studies should further investigate the potential adverse effects of De on focal infections.

Preclinical and clinical data reported on the association of Zo with pro-inflammatory effects and in particular the polarization of M1 macrophages [7, 31], which are of particular relevance to the pathogenesis of MRONJs. Infectious events have also been linked with the administration of Be, a monoclonal antibody targeting the vascular endothelial growth factor A and subsequently angiogenesis in cancer patients. These adverse events were mainly related to severe febrile neutropenia and the occurrence of fistulae/abscesses [32]. The present analysis, however, failed to reveal any apparent effects of either Zo or Be on ICT and CD68 values.

Within its limitations, the present study has indicated that induced peri-implant infections were not associated with any inflammatory lesions in pharyngeal tissues. While these findings were similar under Zo and Be medication, De and De+Be had a marked effect on ICT and CD68 values. The clinical relevance of these findings needs further investigation.

## Conclusions

Within the limitations of an experimental animal study, it was concluded that induced peri-implant infections were not associated with any inflammatory lesions in pharyngeal tissues. While these findings were similar under Zo and Be medication, De and De+Be had a marked effect on ICT and CD68 values. The clinical relevance of these adverse findings needs further investigation.

## Data Availability

The availability of raw data used and/or analyzed during the current study is limited/restricted by general data protection regulations.

## Abbreviations

Be: Antiangiogenic medication Bevacizumab
CD68: Cluster of differentiation 68
De: RANKL inhibitor Denosumab
ICT: Inflammatory cell infiltrate
MRONJ: Medication-related osteonecrosis of the jaw
RANKL: Inhibitors of Receptor Activator of NF-κB Ligand
Zo: Amino-bisphosphonate Zoledronate
